# Increasing hip fracture volume following repeated lockdowns: an Irish multicentre study of periods pre-Covid, during Covid lockdown and following vaccination

**DOI:** 10.1007/s11845-022-03002-0

**Published:** 2022-04-14

**Authors:** Conor S. O’Driscoll, Colum Downey, Charles Timon, David Lennon, Louise Brent, Colin G. Murphy, May Cleary, John F. Quinlan

**Affiliations:** 1 Department of Orthopaedics, Tallaght University Hospital, Dublin 24, Ireland; 2 Galway University Hospital, Galway, Ireland; 3 Waterford University Hospital, Waterford, Ireland; 4 University College Cork, Cork, Ireland; 5 Irish Hip Fracture Database, National Office of Clinical Audit, Dublin, Ireland

**Keywords:** Covid-19, Hip fracture, Lockdown, Sarcopenia, Trauma care, Vaccination

## Abstract

**Background:**

Older age groups were identified as a high-risk cohort for Covid-19 and thus were a focus of lockdown measures enacted internationally. Resultant decreased social mobility and physical activity levels are associated with sarcopenia, which may lead to increased risk of hip fracture upon resuming social integration and physical activities after easing of lockdown restrictions.

**Aims:**

Our aim was to compare the incidence of hip fractures during the period following vaccination with subsequent relaxation of restrictions, to those prior to and during the Covid pandemic.

**Methods:**

A multicentre retrospective cohort study was performed consisting of all patients presenting with a “hip” fracture to 3 regional trauma units over the relevant time periods in 2019, 2020 and 2021. Tallaght, Galway and Waterford University Hospitals are large academic teaching hospitals with a combined mixed urban and rural catchment of over 1 million people.

**Findings:**

Four-hundred-fourteen patients in total were included in the final analysis, with 133 eligible hip fractures observed proceeding to operative treatment across the study period in 2019, 132 in 2020 and 149 in 2021, representing a 12.88% increase. Demographic data revealed similar patient cohorts with respect to age and gender, fracture pattern and treatment.

**Conclusions:**

An increase in hip fracture volume was observed during the period post vaccination with subsequent relaxation of restrictions and increased social mobility, compared to those prior to and during the Covid pandemic. These findings have implications for hospital planning and orthopaedic resourcing as we navigate our way forward past the Covid-19 Pandemic.

## Introduction

A higher incidence of hip fracture has been demonstrated to occur within an older or frail population group, with a mean age of 81 in the 2019 Irish Hip Fracture National Report [1]. This cohort of the population is also particularly vulnerable to Covid-19 pandemic, [2] and were thus the focus of age-based lockdown measures enacted internationally [3]. These restrictions led to decreased social mobility [4] and resultant reductions in physical activity, particularly within the older age cohort [5].

However, lockdown restrictions with reductions in physical activity and changes in dietary intakes have the potential to accelerate sarcopenia and frailty [6]. This may make an already vulnerable elderly population at increased risk of fracture upon resuming social integration and physical activity following the rollout of vaccination programs and easing of lockdown restrictions [7].

Our study aim was to compare the incidence of hip fractures following vaccination with subsequent relaxation of restrictions and increased social mobility, to those prior to and during the Covid pandemic. The time points of April and May were chosen, as this coincided with a period of strict lockdown within Ireland in 2020 [8], while also representing a time point during which over 95% of the over 70 age group had received at least 1 vaccine dose in Ireland in 2021 [9]. This will serve to inform future research into hip fracture incidence, frailty, rehabilitation, and the lasting effects of the Covid-19 pandemic, while also aiding healthcare systems plan and resource future care.

## Methods

A multicentre retrospective cohort study was performed, with reference to STROBE guidelines [10], consisting of all patients presenting with a proximal femoral, “hip”, fracture to 3 regional trauma units over the 2-month period of April and May during the years 2019, 2020, 2021. Tallaght, Galway and Waterford University Hospitals were chosen as our study group, as they are large academic teaching hospitals with a combined mixed urban and rural catchment area of over 1 million people [11]. National Irish Hip Fracture Database data from a total of units was also included for benchmarking purposes for the periods 2019 and 2020.

Following local and national research ethics committee approval, a review of anonymised hip fracture register data compiled by each site pertaining to each distinct time period was performed. This is a prospectively collated register sourced by local hip fracture coordinators using medical records, theatre logs and imaging systems, with published national coverage rates of 99% [1].

Fracture pattern classification and treatment modalities for the three institutions were further validated retrospectively by the authors (COD, CT, DL), using the respective radiology viewing platforms, NIMIS (National Integrated Medical Imaging System) and AGFA PACS and cross referenced with consultant radiologist reports. Instances of viewer discordance were adjudicated by a senior author (CD). This study included operatively managed extracapsular trochanteric AO/OTA type 3.1.A and intracapsular femoral neck AO/OTA type 3.1.B fractures. Exclusion criteria consisted of patients < 60 years of age and stable fracture patterns such as isolated greater trochanter fractures suitable for non-operative treatment.

Demographic (age, gender) and injury (anatomical location, treatment) data relating to hip fracture patients for each time period was sorted using a standardized template (Microsoft Excel), with Stata software version 16.1 (StataCorp, College Station, TX, USA) used for descriptive statistics.

## Results

Four-hundred-fourteen patients in total were included in the final analysis, with 133 eligible hip fractures observed proceeding to operative treatment across the relevant time period in 2019, 132 in 2020 and 149 in 2021.

**Fig. 1:**
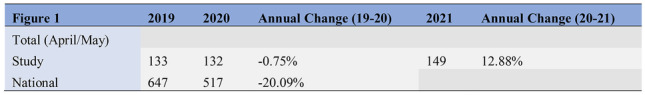
Hip Fracture Volume, 2019/2020/2021. Study and National

**Fig. 2:**
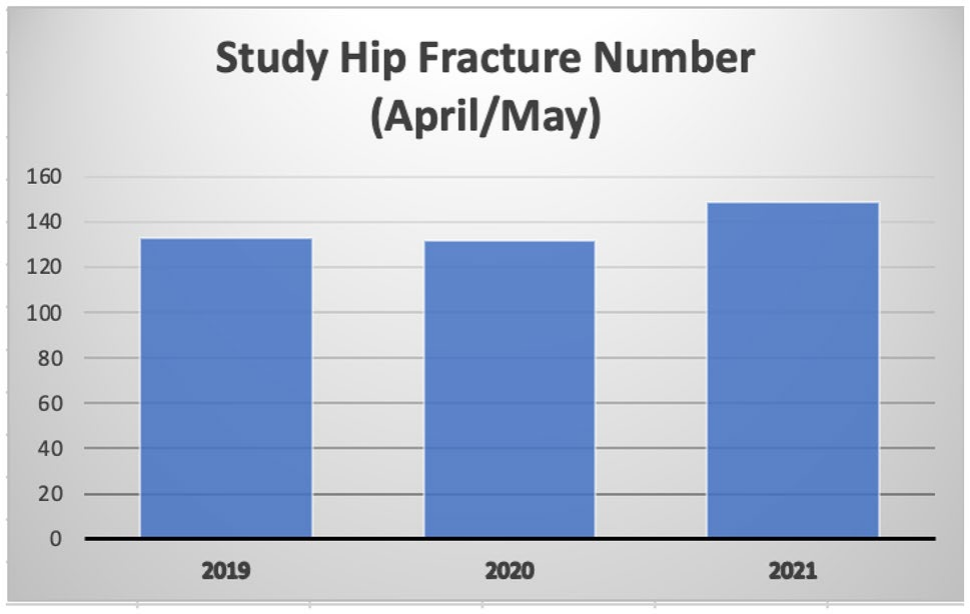
Study Hip Fracture Volume, 2019/2020/2021. Graph

This information is visually displayed in Figs. 1, 2 demonstrating a decline of 0.75% between April/May 2019 compared to 2020. This can be benchmarked against national data, for the same time period where a larger decrease of 20% was reported (see Fig. 1).

There was an increase of 12.88% observed comparing the lockdown period of April/May 2020 against the period of April/May 2021. 

**Fig. 3:**
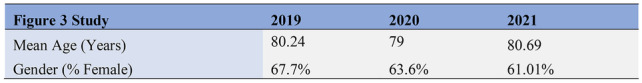
Study demographics (age and gender)

**Fig. 4:**
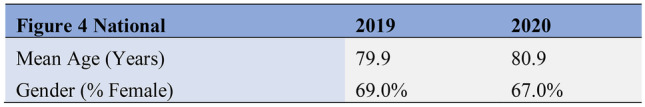
National demographics (age and gender)

**Fig. 5:**
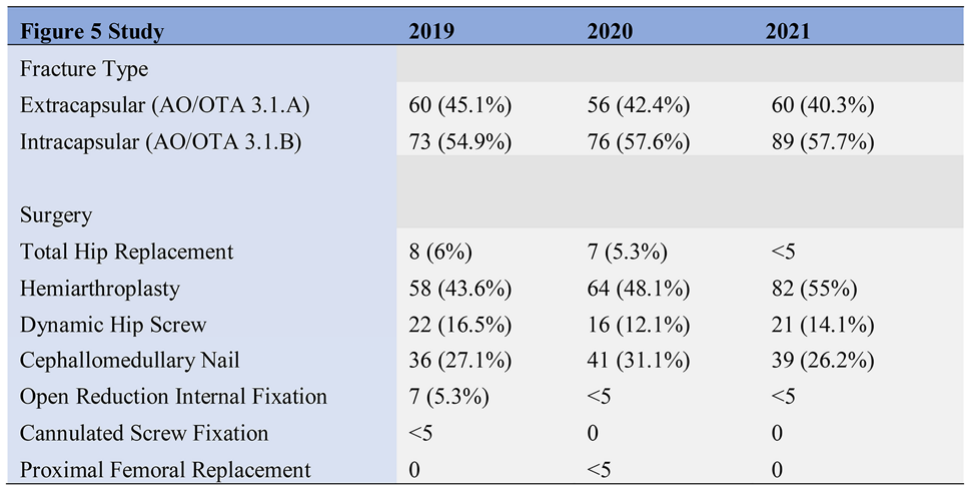
Study fracture classification and treatment

**Fig. 6:**
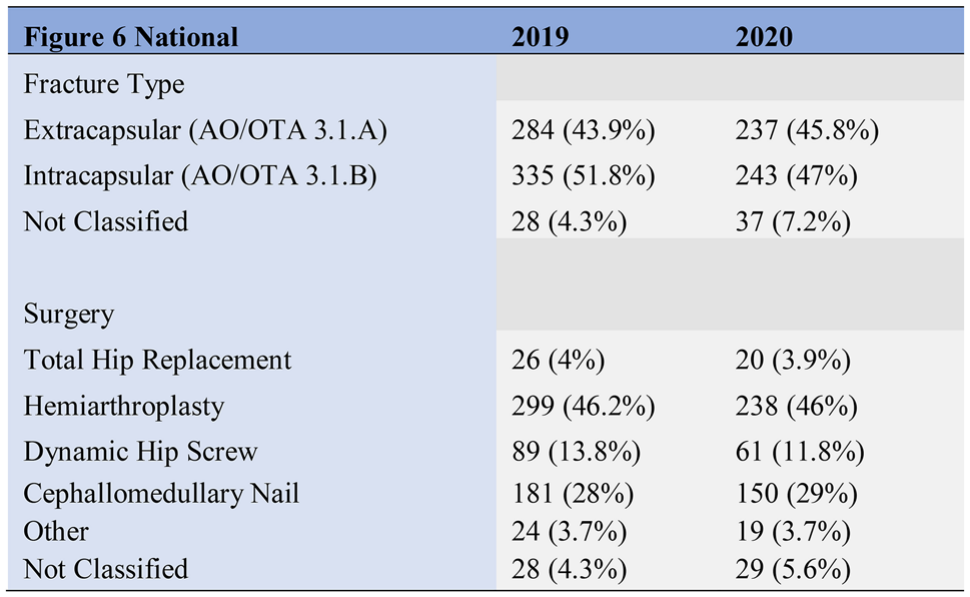
National fracture classification and treatment

Demographic data, as detailed in Figs. 3, 4 below, revealed similar patient cohorts with respect to age and gender. Likewise, fracture pattern and treatment was also consistent across the time points (Figs. 5, 6).

## Discussion

Our results demonstrated no significant change in hip fracture volume across our institutions, between the pre-pandemic control period of April/May 2019 and the period of peak social restrictions in April/May 2020, while nationally, a decrease was observed. Early published reports reported upon a reduced incidence attributed this to imposed lockdown and social restrictions [12, 13]; however, our experience of unchanged hip fracture volume was shared by many units worldwide. A large global survey by Hall et al. [14] of 1855 trauma units across 14 countries worldwide found that 91/173 (52.6%) units reported an unchanged volume of hip fracture admissions with 74/173 (42.8%) reporting a reduction, and 8/173 (4.6%) an increased volume. Hip fractures predominantly occur following low velocity mechanisms such as falls from standing height, commonly occurring in the home environment, and thus did not fully replicate the dramatic fall in major trauma and activity related volume experienced at the beginning of the Covid-19 pandemic [15].

A significant increase in hip fracture volume was observed across our institutions between both the 2019 and 2020 time periods, and April/May 2021. To our knowledge, this was the first study reporting upon hip fracture volume during this time period, and it will be interesting to observe further research which may elucidate possible reasons behind this rise.

Ireland enacted a highly successful vaccination program which resulted in over 95% of the over 70 age group receiving at least one dose by the timepoint of April 2021, and there was a subsequent increase in social mobility and activity within the older age cohort during the period of April and May 2021 [9]. However, repeated lockdown restrictions had been a feature of the preceding year, with 3 separate “waves” of coronavirus cases experienced in Ireland.

An Argentinian orthopaedic unit observed higher levels of frailty across their hip fracture group following lengthy quarantine periods, with a statistically significant rise in proportion of those with a high Rockwood frailty score > 5 of 42% vs 32% despite similar age and gender demographics [7]. Constandt et al. in their survey of 15,737 Belgian adults found that those over 55 years of age self-reported lower levels of exercises during lockdown periods, with similar findings that were replicated across multiple other surveys [5, 16, 17]. Sarcopenia has been shown to have a positive association with falls and fractures in older adults [18], and it follows that decreased physical activity with resultant sarcopenia [6] during repeated periods of lockdown may have contributed towards this increased hip fracture incidence.

Contemporaneous accurate representation of fracture incidence and thus care needs are crucial for healthcare planning [19] and our study will aid in the appropriate upscaling of resources for delivering excellent orthopaedic care to this vulnerable group. The benefits of multidisciplinary team care and fracture liaison services have previously been demonstrated for the secondary prevention of hip fractures [20], and lessons learnt in this setting may play a role in helping to rehabilitate those particularly affected by lockdown measures.

This is of critical importance following the covid pandemic in which orthopaedic care resources were often depleted with reallocation of staff across other sectors. In the aforementioned international survey by Hall et al. [14], it was found that 63% of units reported worse service quality with staff redistribution leading to up to 33% reduced dedicated physiotherapist and orthogeriatric services, in addition to reallocation of inpatient areas and reduced operating access.

A potential limitation of this study is sample size. We sought to mitigate this by pooling data from a number of centres across diverse urban and rural catchment areas; however, larger national datasets may provide higher powered studies in time.

Internationally, there was some variation between the political and societal responses to the covid pandemic. While strict lockdowns were a feature of the early response across Ireland and much of Europe, this was not universally the case. While we observed an increase across our centres, we look forward to reading future reports which may indicate differences in hip fracture incidence trends internationally.

## Conclusion

Our multicentre study observed a large increase in hip fracture incidence within older age cohorts, during the period post vaccination with subsequent relaxation of restrictions and increased social mobility, compared to those prior to and during the Covid pandemic. This followed a period of relative inactivity during repeated lockdown restrictions and has implications for healthcare planning and resourcing as we navigate our way forward past the Covid-19 pandemic.
